# An overview of treatment response rates to various anti-viral drugs in Pakistani Hepatitis B Virus infected patients

**DOI:** 10.1186/1743-422X-8-20

**Published:** 2011-01-15

**Authors:** Liaqat Ali, Muhammad Idrees, Muhammad Ali, Irshad-ur Rehman, Abrar Hussain, Samia Afzal, Sadia Butt, Sana Saleem, Saira Munir, Sadaf Badar

**Affiliations:** 1 Division of Molecular Virology, National Centre of Excellence in Molecular Biology, University of the Punjab, 87 - West Canal Bank Road, Thoker Niaz Baig, Lahore 53700, Pakistan

## Abstract

Hepatitis B virus (HBV) is one of the leading health problem with up to 350 million affected people worldwide including 4.5 million only in Pakistan. It has mortality rate of 0.5 to 1.2 million per year worldwide. Pakistan lies in the endemic region with 3-5% HBV carrier rate in the country. The present article reviews the literature on the treatment response of HBV prevalent in Pakistani population. The average treatment response of Lamivudine and interferon-α is 25.81% and 47.95%, respectively. Peg-Interferon was shown to be not effective against the HBV/HCV (hepatitis C virus)/HDV (hepatitis Delta virus) co-infection. The present study reveals that interferon-α is the most effective therapy available for HBV infection prevalent in Pakistani population. Genotype C & D are the most common HBV genotypes in Pakistan and are associated with increased severity and less response to interferon therapy. This poses a great challenge for physicians and researchers and further studies are needed to describe the outcome of the current therapies recommended against HBV infection in Pakistani population.

## Introduction

Hepatitis B virus (HBV) is a crucial health problem with up to 350 million affected people worldwide [1]. HBV is a member of the *Hepadnaviridae *family with 3.2 kilobase pair DNA genome which is partially double-stranded [2,3]. Pre-s domain surface protein is mediates its attachment to the cell membrane [4].

Several previous studies on the association of the HBV genotypes with disease progression reported that genotypes B and C are correlated with severity of liver dysfunction while high viral loads were observed in patients infected with genotype C [5,6], A, and D [7] in some studies but not in others [8,9]. According to WHO, Pakistan has low HBV infection rates of 3%, while studies from Pakistan are more focused towards the HBV prevalence rate [10,11], epidemiological issues [12], genotyping and its core antigen genetic variability [13].

Approved drugs advised for the treatment of HBV include interferon-α, PEG-interferon and antiviral drugs like lamivuaine, adefovir, dipivoxil, entecavir and telbivudine. Hepatitis B antibodies and the HBV vaccine within 12 hours of birth help to prevent the infection [14]. In Pakistan, there are estimated 4.5 million carriers of HBV with a carrier rate of 3-5%. However, studies are limited describing HBV treatment response in Pakistani population. The present article reviews all the available literature on the treatment response to the available therapies against HBV prevalent in Pakistani population (Table 1).

**Table 1:** An overview of treatment outcome in Pakistani HBV treated patients

Author	Region	Patients (n)	Etiology	Treatment	Duration (weeks)	Results
**Qureshi ***et al: *[19]	Karachi	69	HBeAg, HBV DNA positive patients	100 mg of Lamivudine orally before breakfast till seroconversion	36 months	38% cases were observed to sero-converted.

**Qureshi ***et al*: [19]	Karachi	55	HBV DNA positive (wild type) with delta positive	100 mg of Lamivudine orally before breakfast till seroconversion	36 months	16.4% cases in group 2 sero-converted (Wild type of HBV/HDV co-infected cases have a 16% chance of seroconversion)

**Naeem ***et al: *[31]	Rawalpindi	50	Chronic viral hepatitis B (HBsAg and HBV DNA positive)	5 mega units of recombinant interferon alfa-2b subcutaneously once daily	4-months	HBV DNA was found negative in 44.0% (22) patients while treatment was ceased in three patients due to severe depression.

**Zuberi ***et al*: [21]	Karachi	246	co/super-infection of Hepatitis C and D among patients of HBV	pegylated interferon-a 2a 180 mcg sc weekly	48 weeks	HBV was not cleared in any case

**Zuberi **et al: [32]	Karachi	52	patients of hepatitis B with hepatitis D	Interferon - a 10.0 MIU sc t.i.w.	48 weeks	51.9% patients had suppressed (< 400 copies/ml) HBV DNA levels

**Khokhar ***et al*. [21]	Islamabad	105	positive HBsAg and elevated ALT	lamivudine 100 mg once a day for 12 months	12 months and were followed every 2-3 months with ALT, HBeAg and HBV DNA	HBeAg positive and HBeAg negative patients were found with 23.6% and 80.0% treatment response rate respectively (All the patients were HBsAg positive)

## Anti viral efficacy of lamivudine

A nucleoside analogue called lamivudine, has anti-HBV and anti-HIV properties, was approved by the US-FDA in 1998 for the treatment of HBV infections. It is administered orally with concentration of 100 mg/day [15]. Use of lamivudine is reported to decrease the circulating DNA levels of HBV [16,17] while long term decrease has been achieved after one month's therapy [18]. In Pakistani population, the rate of seroconversion against lamivudine was observed to be 16% in HBV/HDV(Hepatitis delta Virus) co-infected patients when administered for 36 months [19], 23 to 38% in HBV infected patients when administered for 12 months and 36 months, respectively (Table 1) [20,21]. A much higher HBV response rate is being observed in Pakistani population than reported earlier [22]. These studies show that lamivudine monotherapy is a better option for treatment of HBV infection but this therapy require longer durations (up to 36 months) and develop increased resistance (up to 20% after one year and 70% after 5 years of therapy) [23,24], which makes it undesirable for the patients.

Nucleotide and nucleoside analogues are shown to be associated with minimum side effects as compared to the standard INF and PEG-INF therapies. These include short term adversed effects like myopathy, neuropathy and lactic acidosis [25]. These include side effects like abdominal pain, headache, cough, nasopharyngitis, pyrexia, diarrhea and fatigue [26].

## Anti viral efficacy of Peg-Interferon

Interferon is supplemented with polyethylene glycol (PEG) which prolongs its half-life resulting in sustained antiviral response rates. Two types of PEG-IFN have been studied for HBV therapy, PEG-IFN α-2a having a large 40 kDa PEG branched and PEG-IFN α-2b with a 12 kDa PEG molecule [27]. Buster and Janssen [22] reported HBV treatment response of 19-35% by administration of peg-IFN. However, administration of pegylated interferon-α 2a for 4 months failed to treat the HBV, HDV and HCV co-infection in Pakistani population [20] and is associated with adverse effects like depression, ﬂu-like symptoms, neuropsychiatric disorders and suppression of bone marrow [28].

This shows that peg-interferon is not effective against HBV when administered in HBV/HDV/HCV co-infected patients. However, there is no study describing the use of peg-interferon against HBV infected Pakistani population.

## Anti viral efficacy of Interferon-α (IFN-α)

IFN-α was approved in 1992 after being extensively studied. It is reported to increase hepatitis B surface antigen (HBsAg) expression by hepatocytes and inhibit packaging of pre-genomic viral RNA into the core particles [29]. Currently, many different types of interferon are available but available data is limited to support advantage of one therapy to be more effective than the others. However, the response rate of 30-40% was reported by the administration of IFN-α for 6-12 months as compared to 10-20% in controls. Patients with lower ALT and/or higher HBV viral loads and immunosuppressed patients are the risk factors that result in decreased response to IFN treatment. Hepatitic flares are shown to predict sustained virological response during interferon treatment [30]. Naeem and coworkers [31] reported that administration of 5MIU of recombinant IFN-α-2b for four months lead to response in 44% of Pakistani patients. On the other hand, Zuberi and colleagues [32] described an increased treatment response of 51.9% after administration of 10.0 MIU of IFN-α in HBV infected patients for the same period of 16 weeks. IFN-α is also shown to be associated with the development of side effects like insomnia, fatigue, alopecia and anorexia [33,34]. However, further studies on the treatment response and follow up of patients are needed to understand the effectiveness of INF-α against HBV infection in Pakistani population.

## HBV genetic heterogeneity and Antiviral Therapy

HBV has been classified into 9 genotypes (A-I) based on 8% or more inter-group divergence in full length genomic sequence [35-38] and its antiviral treatment response rate is highly affected by genetic variability as well as by various host and viral factors.

The most recent study conducted throughout Pakistan has reported that HBV genotype C is the most prevalent genotype in Pakistan with 26.7% prevalence, followed by genotype B (18%), A (14.3%), D (13%), mixed genotypes (14.6%) and 10.3% were found untypable [38]. While genotypes E (0.6%) and F (1.3%) have been reported recently in Pakistan. A very high prevalence of HBV genotype D (60-100%) were also reported from different regions of Pakistan [39-44,13]. It is well understood that both genotype C & D are less responsive to interferon therapy and associated with more severe disease than genotype A and B [45,46]. Moreover, these genotypes are reported to be less frequently related to HBeAg clearance rates than genotypes A and B when treated with pegylated interferon [47]. Another study revealed that antiviral response rate against IFN-alpha was higher in genotype F as compared to genotypes E and G [48].

The high prevalence of HBV genotype D and C in Pakistani population and its association with increased severity of the disease and resistance to the present therapies demands more consistent preventive measures like mass vaccination and awareness programs at national level.

## Conclusion

This review explains that interferon-α is the most effective available drug against the HBV prevalent in Pakistan with up to 47.95% treatment response rate. Moreover, treatment of the resistant but most prevalent HBV genotypes C and D is a challenge for clinicians and scientists in our region. However, further studies are needed to fully describe the treatment response and the risk factors of the currently recommended therapies against HBV infection especially combination therapy of interferon and lamivudine in Pakistani isolates. These future prospects will enable the virologists to focus drug designing in this part of the world.

## Competing interests

The authors declare that they have no competing interests.

## Authors' contributions

LA and MA reviewed the literature, and wrote the manuscript. MI reviewed the manuscript. IR, AH, SA, SB, SS, SM and SB helped LA & MA in literature review. All the authors read and approved the final manuscript.

## References

[B1] Awan Z Idrees M Rafique S Rehman I Akbar H But S Hepatitis B virus YMDD-motif mutations with emergence of lamivudine-resistant mutants: a threat to recovery Gastroenterology and Hepatology From Bed to Bench 2010 3 108 114

[B2] Lee W Hepatitis B virus infection N Engl J Med 1997 337 1733 1745 10.1056/NEJM199712113372406 9392700

[B3] Ganem D Schneiden RJ Knipe DM, Howely PM, Griffin DE Hepadnaviridae: the viruses and their replication Fields Virology 2001 Philadelphia: Lippincott-Raven 292 2969

[B4] Klingmuller U Schallen H Hepadnavirus infection requires interaction between the viral pre-s domain and a specific hepatocellular receptor J Virol 1993 67 7414 7422 8230462 10.1128/jvi.67.12.7414-7422.1993 PMC238206

[B5] Sugauchi F Chutaputti A Orito E Kato H Suzuki S Ueda R Mizokami M Hepatitis B virus genotypes and clinical manifestation among hepatitis B carriers in Thailand J Gastroenterol Hepatol 2002 17 671 676 10.1046/j.1440-1746.2002.02754.x 12100612

[B6] Ishikawa K Koyama T Masuda T Prevalence of HBV genotypes in asymptomatic carrier residents and their clinical characteristics during long-term follow-up: the relevance to changes in the HBeAg/anti-HBe system Hepatol Res 2002 24 1 10.1016/S1386-6346(02)00017-7 12243786

[B7] Westland C Delaney W Yang H Gibbs C Miller M Wulfsohn MJ Hepatitis B virus genotypes and virologic response in 694 patients in phase III studies of adefovir dipivoxil1 Gastroenterology 2003 125 107 116 10.1016/S0016-5085(03)00700-5 12851876

[B8] Sumi H Yokosuka O Seki N Arai M Imazeki F Kurihara T Kanda T Fukai K Kato M Saisho H Influence of hepatitis B virus genotypes on the progression of chronic type B liver disease Hepatology 2003 37 19 26 10.1053/jhep.2003.50036 12500184

[B9] Orito E Mizokami M Sakugawa H Michitaka K Ishikawa K Ichida T A case-control study for clinical and molecular biological differences between hepatitis B viruses of genotypes B and C. Japan HBV Genotype Research Group Hepatology 2001 33 218 223 10.1053/jhep.2001.20532 11124839

[B10] Khichi GQK Channar MS Prevalence of hepatitis B carriers among children in Bahawalpur urban slums Pak J Med Sci 2000 16 238 241

[B11] Khattak MF Salamat N Bhatti FA Qureshi TZ Seroprevalence of hepatitis B, C and HIV in blood donors in northern Pakistan J Pak Med Assoc 2002 52 398 402 12532573

[B12] Akhtar S Younus M Adil S Hassan F Jafri SH Epidemiologic study of chronic hepatitis B virus infection in male volunteer blood donors in Karachi, Pakistan BMC Gastroenterol 2005 5 26 10.1186/1471-230X-5-26 16086833 PMC1208878

[B13] Abbas Z Muzaffar R Siddiqui A Naqvi SAA Rizvi SAH Genetic variability in the precore and core promoter regions of hepatitis B virus strains in Karachi BMC Gastroenterol 2006 6 20 10.1186/1471-230X-6-20 16863587 PMC1544342

[B14] Gilroy RK Mukherjee S Hepatitis A Kanas Med Centre 2008

[B15] Cammack N Rouse P Marr CL Reid PJ Boehme RE Coates JA Penn CR Cameron JM Cellular metabolism of (-) enantiomeric 2'-deoxy-3'-thiacytidine Biochem Pharmacol 1992 43 2059 2064 10.1016/0006-2952(92)90162-C 1318048

[B16] Dienstag JL Schiff ER Wright TL Perrillo RP Hann HW Goodman Z Lamivudine as initial treatment for chronic hepatitis B in the United States N Engl J Med 1999 341 1256 1263 10.1056/NEJM199910213411702 10528035

[B17] Lai CL Chine RW Leung NWY Chang TT Guan R Tai DI A one year trial of lamivudine for chronic hepatitis B N Engl J Med 1998 339 61 68 10.1056/NEJM199807093390201 9654535

[B18] Yuen MF Fong DY Wong DK Yuen JC Fung J Lai CL Hepatitis B virus DNA levels at week 4 of lamivudine treatment predict the 5-year ideal response Hepatology 2007 46 1695 1703 10.1002/hep.21939 18027877

[B19] Qureshi H Arif A Alam E Treatment of HBV and HDV co-infection using lamivudine J Ayub Med Coll Abbottabad 2009 21 1 3 20524456

[B20] Zuberi BF Afsar S Quraishy MS Triple Hepatitis: Frequency and Treatment Outcome of co/super-Infection of Hepatitis C and D Among Patients of Hepatitis B J Coll Physicians Surg Pak 2008 18 404 407 18760062

[B21] Khokhar N Gill ML Alam AY Treatment of chronic Hepatitis B with Lamivudine J Coll Physicians Surg Pak 2005 15 78 80 15730830

[B22] Buster EHCJ Janssen HLA Antiviral treatment for chronic hepatitis B virus infection-immune modulation or viral suppression? The Journal of Medicine 2006 64 175 185 16788215

[B23] Lok AS Lai CL Leung N Yao GB Cui ZY Schiff ER Long-term safety of lamivudine treatment in patients with chronic hepatitis B Gastroenterology 2003 125 1714 1722 10.1053/j.gastro.2003.09.033 14724824

[B24] Pawlotsky JM Dusheiko G Hatzakis A Lau D Lau G Liang TJ Virologic monitoring of hepatitis B virus therapy in clinical trials and practice: recommendations for a standardized approach Gastroenterology 2008 134 405 415 10.1053/j.gastro.2007.11.036 18242209 PMC2676233

[B25] Sonneveld MJ Janssen HLA Pros and cons of peginterferon versus nucleos(t)ide analogues for treatment of chronic hepatitis B Curr Hepatitis Rep 2010 9 91 98 10.1007/s11901-010-0041-7 PMC2861769 20461129

[B26] Chang T Gish RG de-Man R Gadano A Sollano J Chao Y A Comparison of Entecavir and Lamivudine for HBeAg-Positive Chronic Hepatitis B N Engl J Med 2006 354 1001 10 10.1056/NEJMoa051285 16525137

[B27] Craxi A Cooksley WG Pegylated interferons for chronic hepatitis B Antiviral Res 2003 60 87 89 10.1016/j.antiviral.2003.08.015 14638403

[B28] Chang T Suh DJ Current approaches for treating chronic hepatitis B: when to start, what to start with, and when to stop Hepatol Int 2008 2 S19 S27 10.1007/s12072-008-9059-0 PMC2716841 19669295

[B29] Lau JYN Bain VG Naomov NV Smith HM Alexander GJ Williams R Effect of interferon-a on hepatits B viral antigen expression in primary hepatocyte culture Hepatology 1991 14 9759 1959885

[B30] Guan R Interferon monotherapy in chronic hepatitis B J Gastroenterol Hepatol 2000 15 34 40 10.1046/j.1440-1746.2000.02101.x 10921380

[B31] Naeem MA Khan I Waris J Usman M Efficacy of Interferon therapy in patients of chronic hepatitis B viral infection treated at MH Rawalpindi Professional Med J 2008 15 380 386

[B32] Zuberi BF Quraishy MS Afsar S Akhtar N Kumar A Dodani SK Treatment outcome in patients of hepatitis B with hepatitis D: Experience of 4 years at a tertiary care centre in Pakistan J Coll Physicians Surg Pak 2007 17 320 322 17623577

[B33] Okushin H Ohnishi T Morii K Uesaka K Yuasa S Short-term intravenous interferon therapy for chronic hepatitis B World J Gastroenterol 2008 14 3038 3043 10.3748/wjg.14.3038 18494055 PMC2712171

[B34] Cooksley WG Piratvisuth T Lee SD Mahachai V Chao YC Tanwandee T Chutaputti A Chang WY Zahm FE Pluck N Peginterferon alpha-2a (40 kDa): an advance in the treatment of hepatitis B e antigen-positive chronic hepatitis B J Viral Hepat 2003 10 298 305 10.1046/j.1365-2893.2003.00450.x 12823597

[B35] Okamoto H Tsuda F Sakugawa H Sastrosoewinjo RI Imai M Miyakawa Y Mayumi M Typing hepatitis B virus by homology in nucleotide sequence: comparison of surface antigen subtypes J Gen Virol 1988 69 2575 2583 10.1099/0022-1317-69-10-2575 3171552

[B36] Huy T Ngoc T Abe K New complex recombinant genotype of hepatitis B virus identified in Vietnam J Virol 2008 82 5657 5663 10.1128/JVI.02556-07 18353958 PMC2395174

[B37] Yu H Yuan Q Ge SX Wang HY Zhang YL Chen QR Zhang J Chen PJ Xia NS Molecular and phylogenetic analyses suggest an additional.Hepatitis B virus genotype ''I'' PLoS One 2010 5 9297 10.1371/journal.pone.0009297 PMC2824819 20174575

[B38] Awan Z Idrees M Amin I Pattern and Molecular Epidemiology of Hepatitis B virus genotypes circulating in Pakistan Infect Genet Evol 2010 10 1242 1246 10.1016/j.meegid.2010.08.006 20727423

[B39] Ahmed CS Wang ZH Bin Z Chen JJ Kamal M Hou JL Hepatitis B virus genotypes, subgenotypes, precore, and basal core promoter mutations in the two largest provinces of Pakistan J Gastroenterol Hepatol 2009 24 569 73 10.1111/j.1440-1746.2008.05642.x 19368634

[B40] Baig S Siddiqui A Chakravarty R Moatter T Hepatitis B virus subgenotypes D1 and D3 are prevalent in Pakistan BMC Research Notes 2009 2 1 10.1186/1756-0500-2-1 19121226 PMC2636821

[B41] Noorali S Hakim ST McLean D Kazmi SU Bagasra O Prevalence of Hepatitis B virus genotype D in females in Karachi, Pakistan J Infect Developing Countries 2008 2 373 378 10.3855/jidc.200 19745506

[B42] Hakim ST Kazmi SU Bagasra O Seroprevalence of hepatitis B and C genotypes among yung apparently healthy females of Karachi-Pakistan Libyan J Med 2008 3 66 70 AOP: 071123 10.4176/071123 PMC3074282 21499460

[B43] Alam MM Zaidi SZ Shaukat S Sharif S Angez M Naeem A Common Genotypes of Hepatitis B virus prevalent in Injecting drug abusers (addicts) of North West Frontier Province of Pakistan Virology J 2007 4 63 10.1186/1743-422X-4-63 PMC1910598 17597548

[B44] Baig S Siddiqui AA Ahmed W Qureshi H Arif A The association of complex liver disorders with HBV genotypes prevalent in Pakistan Virology J 2007 4 128 10.1186/1743-422X-4-128 PMC2212638 18042293

[B45] Kao JH Wu NH Chen PJ Lai MY Chen DS Hepatitis B genotypes and the response to interferon therapy J Hepatol 2000 33 998 1002 10.1016/S0168-8278(00)80135-X 11131465

[B46] Wai CT Chu CJ Hussain M Lok AS HBV genotype B is associated with better response to interferon therapy in HBeAg-positive chronic hepatitis than genotype C Hepatology 2002 36 1425 30 12447868 10.1053/jhep.2002.37139

[B47] Janssen HLA Senturk H Zeuzem S Akarka U Cakalluglu Y Simon K Peginterferon alfa 2b and lamivudine combination therapy compared withpeginterferon alfa 2b for chronic HBeAg positive hepatitis B: randomized controlled trial in 307 patients Hepatology 2003 38 1323

[B48] Erhardt A Gobel T Ludwig A Response to antiviral treatment in patients infected with hepatitis B virus genotypes E-H J Med Virol 2009 81 1716 1720 10.1002/jmv.21588 19697400

